# Association of providing/receiving support on the mortality of older adults with different living arrangements in Taiwan: a longitudinal study on ageing

**DOI:** 10.1017/S0144686X17000484

**Published:** 2017-06-29

**Authors:** MIAO-YU LIAO, CHIH-JUNG YEH, SHU-HSIN LEE, CHUN-CHENG LIAO, MENG-CHIH LEE

**Affiliations:** * Institute of Medicine, Chung Shan Medical University, Taichung, Taiwan, ROC.; † Department of Family Medicine, Taichung Hospital, Ministry of Health and Welfare, Taiwan, ROC.; ‡ School of Public Health, Chung Shan Medical University, Taichung, Taiwan, ROC.; § School of Nursing, Chung Shan Medical University, Taichung, Taiwan, ROC.; || Department of Family Medicine, Taichung Armed Forces General Hospital, Taiwan, ROC.; ¶ School of Medicine, National Defense Medical Center, Taipei, Taiwan, ROC.; ** Institute of Population Health Sciences, National Health Research Institutes, Miaoli, Taiwan, ROC.; †† College of informatics, Chao-Yung University of Technology, Taichung, Taiwan, ROC.; ‡‡ School of Public Health, National Defense Medical Center, Taipei, Taiwan, ROC.

**Keywords:** living arrangement, mortality, providing family support, Taiwan Longitudinal Study on Aging

## Abstract

This longitudinal study evaluated the direct effects of providing/receiving family support on mortality in older adults with different living arrangements in Taiwan. All data analysed were obtained from the Taiwan Longitudinal Study on Aging, 1996–2007, of residents aged ⩾67 years (1,492 men and 1,177 women) and Taiwan's National Death Register. Living arrangements were divided into living alone, living only with spouse, living with family and living with others. Support was mainly defined as family support divided into two categories: providing and receiving. The effect of providing/receiving family support on the mortality of older adults was evaluated using Cox regression analysed by living arrangement. Participants living with their families had lower educational levels (illiterate or elementary school) and more disability in both activities of daily living and instrumental activities of daily living. However, they provided more family support than those in other living arrangements. After adjusting for several potentially confounding variables, including background characteristics, economic status and various health status measures, results showed that older adults living with their families and providing support had an 11 per cent lower mortality rate (Hazard ratio = 0.89; 95 per cent confidence interval = 0.83–0.96; *p* = 0.0018). In conclusion, we found that, when living with family, the lives of older adults can be extended by providing support, clearly supporting the old adage ‘it is more blessing to give than to receive’. Older adults wanting to extend their lives can be encouraged to provide more help to their families.

## Introduction

Population ageing is a worldwide phenomenon. The population of older adults 65 years and older in Taiwan was about three million at the end of 2015. People this age make up about 12.8 per cent of the total population there (Department of Health 2016). Population ageing has been accelerating and will accelerate faster in the near future. It is estimated that it will take only 25 years to double from 7 per cent in 1993 to 14 per cent in 2018 in Taiwan, and will take only eight years to increase from 14 to 20 per cent, a rate much faster than many Western countries (National Development Council 2016).

During this accelerated ageing period in Taiwan, society there has rapidly transformed from an agricultural one to an industrial one. Family structure also experienced rapid changes (Fricke, Chang and Yang 1994; Hermalin, Liu and Freedman 1994). In the traditional agriculture society in Taiwan, it was common for three generations of family to live together in one residence, thus caring for older people was less of a problem to the health system because their families cared for them. However, nuclear families (two generations – parents with their unmarried children) have become a more prevalent living arrangement in the Taiwanese society. According to a recent study in Belgium, living arrangement is more associated with mortality than marital status (Herm, Anson and Poulain 2016). Therefore, it would be interesting to investigate the effect of living arrangements on mortality in people of Han Chinese descent living in Taiwan.

In Taiwan, not only do the new living arrangements put older people at a disadvantage, so does their lack of education. Most older adults have a very low level of education because they lacked educational opportunity when they were young, especially women during the Japanese colonial period prior to the Second World War. Survey results show that about half of older men and about three-quarters of older women are either illiterate or have less than primary school education. This lack of education puts them at a disadvantage when it comes to their own care as they must fill out forms, and understand medical directions and educational material, *etc*. This issue deserves research attention because the public network of care for older people may not have kept up with the needs created by accelerated ageing and changes in family structure.

There is an old saying in Taiwan, ‘It is more a blessing to give than to receive’ and in the West ‘It is better to give than receive’. However, there is no clear empirical evidence supporting this old saying particularly with regard to longevity in older adults. Previous research has focused on the association between social support, including family support as a whole, and mortality among older adults (Blazer 1982; Mazzella *et al*. 2010). It has been argued that the influence of social support can be further categorised into two non-mutually exclusive groups: those that are providing social support and those who are receiving social support (Tardy 1985). A European study investigating the association between having grandchildren and health found a significant positive association between the two in grandmothers alone (Gessa, Glaser and Tinker 2016). If a grandmother's role is more likely to be one of providing additional care to grandchildren, then their provision of care may confer upon them some health and longevity benefits. Poulin *et al.* (2013) found that helping others can reduce mortality by buffering the association between stress and mortality. Seeman *et al.* (2002) have suggested that providing support is good for immune, endocrine and cardiovascular function, and can reduce allostatic load. To date, there has been no published study investigating the impact of providing or receiving social support on the mortality of elderly individuals residing in different living arrangements in Asia, in this case, Taiwan.

In this study, we hypothesised that older adults would have a significantly lower mortality risk and eventually have longer survival if they provided support and help to their family members rather than just receiving support only from their family members.

## Participants and methods

### Study design and data collection

Data were extracted from the Taiwan Longitudinal Study on Aging (TLSA), a longitudinal survey of a nationally representative sample conducted by the Bureau of Health Promotion at Taiwan's Department of Health in collaboration with the Population Studies Centre and the Institute of Gerontology at the University of Michigan in the United States of America.

The data from this study were based on six TLSA interviews of the same participants over an eight-year period. The cohort, conducted in 1989, was a national representative sample of older adults aged ⩾60 years residing in Taiwan, including those at home and in institutions. Personal interviews were performed by trained interviewers. In total, 4,049 participants completed this first survey in 1989. They were re-interviewed five more times over the subsequent eight years: in 1993, 1996, 1999, 2003 and 2007. Data collected in the 1996 follow-up survey for those aged 67 and older were used as baseline data in our prediction of mortality over the next 11 years from 1996 to 2007. Details of the TLSA design have been described elsewhere (Lee *et al*. 2012; Liao *et al*. 2011; Yen *et al*. 2010).

### Dependent variable: mortality

This study examined the direct or net effect of receiving and giving family support on mortality of older adults in Taiwan. Therefore, mortality was the only dependent variable. It was measured in survival years estimated starting from 1996 to 2007 using Taiwan's National Death Registration Record which provides survival status and date of death. The participants’ national identification number was used to link the data from the two databases at each of the follow-up surveys.

### Independent variables: providing and receiving family support

The support scale in the 1996 survey was divided into two categories: providing and receiving family support. The concept of providing/receiving support was modified from previous articles (Katz *et al*. 1963; Liang, Krause and Bennett 2001). All items assessed were adopted from the TLSA questionnaire.

The level of family support received was assessed by scoring the responses to the following four family support-related questions: (a) ‘Can you rely on your family and/or relatives for help when you are sick?’, (b) ‘Are you satisfied with your family's and/or relatives’ help when you need help?’, (c) ‘Are your family and/or relatives willing to listen to you when you need to talk?’ and (d) ‘Do you feel that your family and/or relatives care about you?’ Raw Cronbach alpha of these four questions was 0.8384. The level of support the participant provided his or her family was assessed by scoring responses to the following three related questions: (a) ‘Do you wonder if your family members or relatives will come to talk to you when they have troubles or difficulties?, (b) ‘Do your family members and/or relatives seek your opinions?’ and (c) ‘How much help do you think you provide to your family members and/or relatives?’ Raw Cronbach alpha of these three questions was 0.7439.

### Control variables: background characteristics, family income and health status

As shown in Table 1, we also collected the following participant information from the 1996 survey: age (classified into three age groups of 67–69, 70–74 and ⩾75), sex, area of residence (urban, suburban or rural), ethnicity (Fukien, Hakka, Mainlander or others), education level (illiterate: 0 years; elementary school: 1–6 years; junior high school to senior high school: 7–12 years; college: >12 years) and family income (categorised into self-assessed adequate and inadequate).

**Table 1.** Socio-demographic data and health characteristics of the Taiwanese elderly population in 1996, from the Taiwan Longitudinal Study on Aging

Variables	N (%)
Age in 1996 (N = 2,668):	
67–69	688 (25.8)
70–74	996 (37.3)
⩾75	984 (36.9)
Sex (N = 2,668):	
Male	1,492 (55.9)
Female	1,176 (44.1)
Type of area (N = 2,643):	
Urban	995 (37.6)
Suburban	596 (22.6)
Rural	1,052 (39.8)
Ethnicity (N = 2,659):	
Fukienese	1,631 (61.3)
Hakka	409 (15.4)
Mainlander	583 (21.9)
Others	36 (1.4)
Education level (N = 2,668):	
Illiterate	1,047 (39.2)
Elementary school	1,100 (41.2)
Junior high to senior high school	381 (14.3)
College and above	140 (5.3)
Income (N = 2,403):	
Adequate	983 (40.9)
Inadequate	1,420 (59.1)
Living arrangement (N = 2,668):	
Living alone	305 (11.4)
Living only with spouse	455 (17.1)
Living with family	1,868 (70.0)
Living with others	40 (1.5)
Major diseases (N = 2,661):	
None	1,395 (52.4)
At least one	1,266 (47.6)
Self-rated health (N = 2,419):	
Fair and above	1,595 (65.9)
Poor	824 (34.1)
CES-D scale (N = 2,389):	
<10 (no depression)	1,746 (73.1)
⩾10 (depression)	643 (26.9)
IADL disability (N = 2,644):	
None	1,373 (51.9)
At least one	1,271 (48.1)
ADL disability (N = 2,667):	
None	2,392 (89.7)
At least one	275 (10.3)
Receiving family support^1^ (N = 2,309)	11.66 ± 3.30
Providing family support^1^ (N = 2,300)	3.59 ± 2.19

*Notes*: CES-D: Center for Epidemiologic Studies Depression. IADL: instrumental activities of daily living. ADL: activities of daily living. 1. Mean ± standard deviation.

Health status-related measures in 1996 included major diseases, depression measures based on the ten-item Center for Epidemiologic Studies Depression (CES-D) scale, self-rated health and functional disabilities (activities of daily living (ADLs) and instrumental activities of daily living (IADLs)). Major diseases included five physician-diagnosed chronic-based diseases that may affect mortality (cancer, cardiovascular diseases, stroke, diabetes and hypertension). These were combined into one measure and assessment was divided into *none* and *at least one* major disease. Depression was assessed using the ten items of CES-D scale (Boey 1999), providing a score range from 0 to 30. A score of 10 or more was used as the cut-off point to define *having* or *not having* depression (Lee *et al*. 2012). Self-rated health was also divided into two groups: *fair and above* and *poor*. Functional disability was assessed primarily according to the IADL scale which include the participants’ ability to shop, manage money, take a bus or train alone, do light housekeeping, indoor or nearby heavy work, and use the telephone. These were combined into one and assessment was divided into *no difficulty* and *at least one difficulty* (Lawton and Brody 1969). The ADL scale was defined based on the definition of Katz *et al*. (1963). It included bathing, dressing, toileting, transferring, continence and eating. These were combined into one assessment divided into *no difficulty* and *at least one difficulty*.

### Living arrangements

The older adults were further stratified by their living arrangements at the time of 1996 survey. Living arrangements were stratified into four groups: (a) living alone, (b) living only with spouse, (c) living with family including living with spouse as well as married children, married son and his wife, married daughters or grandchildren, and (d) living with others including those living at institutions or living with other relatives not included in family relations above or non-relatives. People living with a skipped generation were classified as living with family because of their relatively small numbers.

### Statistical methods

For independent variables, background characteristics, economic status and health status-related measures data as well as living arrangement were analysed descriptively as numbers with percentages and mean ± standard deviation. This study investigated the direct effect of providing and receiving family support on mortality of older adults. We adjusted for certain background characteristics, economic status and various health status measures in our multivariate Cox regression analysis because these variables may confound mortality and the two independent variables. Analytic results for Models 1 and 2 can be seen Table 3. In Model 1, only background characteristics and economic status are controlled. In Model 2, all background characteristics and economic status as well as several heath status measures are controlled. We further stratified the results by living arrangements to examine the difference of the direct effect in different living arrangements.

## Results

### The background and health characteristics of participants

As can be seen Table 1, a summary of the socio-demographic background characteristics data and health characteristics of the participants in 1996, 37.3 per cent of the participants were between the ages of 70 and 74 years, 55.9 per cent were female and most (39.8%) lived in rural areas. More than 60 per cent (61.3%) were Fukienese, and 80.5 per cent had less than six years of education (including illiteracy or elementary school education). More than half (59.1%) felt that their income level was inadequate. Most (70.0%) lived with their families and 47.6 per cent had at least one major disease. More than 60 per cent (65.9%) believed their health conditions to be fair and above. Nearly 73.1 per cent did not have depressive symptoms (CES-D scale score <10), 48.1 per cent had at least one IADL disability and 10.3 per cent had at least one ADL disability. The average score of those receiving family support from others was 11.66 ± 3.30 (range 0–16), and that of those providing family support was 3.59 ± 2.19 (range 0–7).

### Correlated factors of elderly Taiwanese people's living arrangements in 1996

As shown in Table 2, those living with a spouse only were younger and had a longer average follow-up. Those living with their families tended to be women living in urban areas. Participants living alone or living with others were more likely to be depressed. Participants who lived with families with lower educational levels (illiterate or elementary school) had more IADL and ADL disabilities than those in other types of living arrangement.

**Table 2.** Correlated factors of elderly Taiwanese people's living arrangements in 1996, from the Taiwan Longitudinal Study on Aging

Variables	Living only with spouse	Living with family	Living alone	Living with others	Total N (%)	*p*
	*Frequencies (%) or Mean* ± *standard deviation*					
Average age in 1996 (N = 2,668)	72.72 ± 4.56	73.92 ± 5.64	74.05 ± 5.48	75.60 ± 5.94		<0.0001
Average years of follow-up (N = 2,668)	8.69 ± 3.76	8.24 ± 3.80	7.94 ± 3.87	6.36 ± 4.19		0.0005
Age in 1996 (N = 2,668):						0.0159
67–69	135 (29.7)	475 (25.4)	71 (23.3)	7 (17.5)	688 (25.8)	
70–74	184 (40.4)	689 (36.9)	110 (36.1)	13 (32.5)	996 (37.3)	
⩾75	136 (29.9)	704 (37.7)	124 (40.6)	20 (50.0)	984 (36.9)	
Sex (N = 2,668):						<0.0001
Male	166 (36.5)	886 (47.4)	108 (35.4)	16 (0.4)	1,176 (44.1)	
Female	289 (63.5)	982 (52.6)	197 (64.6)	24 (0.6)	1,492 (55.9)	
Area (N = 2,643):						<0.0001
Urban	139 (30.7)	731 (39.6)	99 (32.7)	26 (65.0)	995 (37.6)	
Suburban	79 (17.4)	437 (23.7)	71 (23.4)	9 (22.5)	596 (22.6)	
Rural	235 (51.9)	679 (36.7)	133 (43.9)	5 (12.5)	1,052 (39.8)	
Ethnicity (N = 2,659):						<0.0001
Fukienese	265 (58.2)	1217 (65.3)	128 (42.4)	21 (52.5)	1,631 (61.3)	
Hakka	75 (16.5)	294 (15.8)	36 (11.9)	4 (10.0)	409 (15.4)	
Mainlander	108 (23.8)	325 (17.5)	135 (44.7)	15 (37.5)	583 (21.9)	
Others	7 (1.5)	26 (1.4)	3(1.0)	0 (0.0)	36 (1.4)	
Education level (N = 2,668):						<0.0001
Illiterate	137 (30.1)	788 (42.2)	108 (35.4)	14 (35.0)	1,047 (39.2)	
Elementary school	193 (42.4)	749 (40.1)	140 (45.8)	18 (45.0)	1,100 (41.2)	
Junior high to senior high school	82 (18.0)	250 (13.4)	42 (13.8)	7 (17.5)	381 (14.3)	
College and above	43 (9.5)	81 (4.3)	15 (5.0)	1 (2.5)	140 (5.3)	
Income (N = 2,403):						0.7902
Adequate	174 (40.1)	684 (41.5)	114 (39.3)	11 (35.5)	983 (40.9)	
Inadequate	260 (59.9)	964 (58.5)	176 (60.7)	20 (64.5)	1,420 (59.1)	
Major diseases (N = 2,661):						0.3028
None	250 (54.9)	963 (51.7)	165 (54.5)	17 (42.5)	1,395 (52.4)	
At least one	205 (45.1)	900 (48.3)	138 (45.5)	23 (57.5)	1,266 (47.6)	
Self-rated health (N = 2,419):						0.4578
Fair and above	287 (65.7)	1,108 (66.7)	180 (62.1)	20 (62.5)	1,595 (65.9)	
Poor	150 (34.3)	552 (33.3)	110 (37.9)	12 (37.5)	824 (34.1)	
CES-D scale (N = 2,389)						0.0002
<10 (no depression)	332 (76.7)	1,211 (73.9)	187 (65.4)	16 (51.6)	1,746 (73.1)	
⩾10 (depression)	101 (23.3)	428 (26.1)	99 (34.6)	15 (48.4)	643 (26.9)	
IADL disability (N = 2,644)						<0.0001
None	281 (62.0)	897 (48.4)	181 (60.5)	14 (35.0)	1,373 (51.9)	
At least one	172 (38.0)	955 (51.6)	118 (39.5)	26 (65.0)	1,271 (48.1)	
ADL disability (N = 2,668):						<0.0001
None	426 (93.6)	1,664 (89.1)	278 (91.1)	24 (60.0)	2,392 (89.7)	
At least one	29 (6.4)	203 (10.9)	27 (8.9)	16 (40.0)	275 (10.3)	
Receiving family support (N = 2,309)	12.06 ± 3.02	11.94 ± 3.07	9.52 ± 4.07	10.32 ± 3.81		<0.0001
Providing family support (N = 2,300)	3.84 ± 2.13	3.67 ± 2.15	2.47 ± 2.23	2.29 ± 2.35		<0.0001

*Notes*: CES-D: Center for Epidemiologic Studies Depression. IADL: instrumental activities of daily living. ADL: activities of daily living.

### Multivariate Cox regression model predictors of all-cause mortality: stratified by living arrangement

Table 3 shows the association between receiving or providing family support and mortality, with participants stratified by living arrangement in the multivariate Cox regression model after adjusting several covariates. In Model 1, we adjusted all social demographic background characteristic variables including age, sex, ethnicity, education level and income. In Model 2, we adjusted all confounding variables, including all background characteristics, income and all health status measures, such as major diseases, self-rated health, CES-D score, and IADL and ADL disabilities. We found a significant association in Model 1 (Hazard ratio (HR) = 0.82; 95% confidence interval (CI) = 0.77–0.88; *p* < 0.0001) and in Model 2 (HR = 0.89; 95% CI = 0.83–0.96; *p* = 0.0018) between providing family support and reduced mortality risk in participants living with their families. No significant association was found between providing or receiving family support and mortality in the group who lived only with spouse or alone. The number of participants living with others (non-relatives) was too small to include in the stratified analysis.

**Table 3.** The multivariate Cox regression model predictors of all-cause mortality: stratified by living arrangement

Variables	Living only with spouse				Living with family				Living alone			
	Model 1		Model 2		Model 1		Model 2		Model 1		Model 2	
	HR	*p*	HR	*p*	HR	*p*	HR	*p*	HR	*p*	HR	*p*
Family support variables:												
Providing family support	0.86	0.0687	0.91	0.2885	0.82	<0.0001	0.89	0.0018	0.97	0.7065	1.06	0.5539
Receiving family support	0.91	0.3062	0.97	0.6997	0.98	0.6072	0.97	0.4526	0.94	0.3326	0.99	0.9023
Controlled confounders:												
Background characteristics:												
Age	1.09	<0.0001	1.09	<0.0001	1.09	<0.0001	1.09	<0.0001	1.08	<0.0001	1.08	<0.0001
Sex	1.73	0.0013	2.06	<0.0001	1.71	<0.0001	2.04	<0.0001	1.94	0.0013	2.54	<0.0001
Suburban *versus* urban	1.22	0.3706	1.21	0.4163	1.09	0.3557	1.08	0.3981	0.80	0.3233	0.74	0.2120
Rural *versus* urban	1.07	0.7131	1.10	0.6212	1.08	0.3877	1.07	0.4262	1.13	0.5551	1.20	0.4023
Hakka *versus* Fukienese	1.12	0.5516	1.07	0.7375	1.01	0.9032	1.02	0.8232	0.72	0.2157	0.55	0.0486
Mainlander *versus* Fukienese	0.76	0.2122	0.71	0.1119	0.79	0.039	0.77	0.0255	0.82	0.3365	0.81	0.3295
Others *versus* Fukienese	1.67	0.3247	1.66	0.3281	1.11	0.7174	1.12	0.712	0.79	0.8198	0.89	0.9113
Elementary school *versus* illiterate	1.06	0.7329	1.09	0.6472	0.96	0.6314	1.00	0.9636	1.07	0.7385	1.00	0.9897
Junior to senior high school *versus* illiterate	0.93	0.7689	1.11	0.6834	0.76	0.0491	0.82	0.156	0.77	0.3757	0.63	0.1493
College *versus* illiterate	0.75	0.3901	0.92	0.8217	0.72	0.1361	0.75	0.1798	1.86	0.1009	1.84	0.1123
Income	0.73	0.0485	0.84	0.3149	0.93	0.3355	0.96	0.5861	0.99	0.9592	1.01	0.9677
Health-related variables:												
Major diseases			1.21	0.2274			1.28	0.001			1.10	0.5894
Self-rated health			0.69	0.0331			0.91	0.2607			0.72	0.1155
CES-D scale			1.28	0.1857			1.19	0.0537			0.96	0.8498
IADL disability			1.38	0.0777			1.32	0.0018			1.02	0.9192
ADL disability			0.98	0.9545			2.03	<0.0001			3.53	0.0007

*Notes*: HR: Hazard ratio. CES-D: Center for Epidemiologic Studies Depression. IADL: instrumental activities of daily living. ADL: activities of daily living.

## Discussion

In this study using data from the TLSA, we assessed the direct impact of providing and receiving support in various living arrangement on the mortality of older adult individuals in Taiwan and found providing family support to be associated with 18 per cent improved mortality in Model 1 (adjusted only for social demographic background characteristics) and 11 per cent improved mortality in Model 2 (adjusted for social demographics and health-related covariates) in older Taiwanese people living with their families. Although the impact of social support has been previously evaluated with regard to the wellbeing of elderly individuals, no study to date has evaluated the impact of receiving and providing family support on overall mortality in ageing individuals.

This study found people living with their family members had lower educational levels and greater IADL and ADL disabilities than those in other living arrangements. However, they were found to provide more family support. Providing more family support may help offset the effect of poor health on mortality risk of those living with their family members. These results confirmed those of Brown *et al*. (2003), who found that providing support was more advantageous than receiving it. Providing family support may generate positive emotions, which have been shown to improve cardiovascular system functioning, and may have a positive effect on the immune and neuroendocrine systems (Fredrickson *et al*. 2000; Seeman 1996). Although the education level of the participants living with their families was lower than their peers, providing family support reduced their mortality, a finding similar to that of Katz *et al*. (1963).

This study found that in Taiwan 11.4 per cent of older adults lived alone, 17.1 lived with their spouses and 70.0 per cent lived with their families. These findings were very similar to those shown for individuals for whom data were collected in the year 2000 for the Survey of Living Conditions of older adults in Taiwan by the Ministry of Interior Affairs, which reported 67.8 per cent of people 65 years or older were living with their children, 15.1 per cent were living only with a spouse and 9.2 per cent were living alone. That survey accounted for 92.1 per cent of Taiwan's older population (Ministry of Interior Affairs 2000). The results of these two studies are consistent with Taiwan's traditional mores which encourage older adults to live with their children or grandchildren and care for one another. In fact, Asian countries frequently lack a comprehensive elderly care social welfare system, which leads to most people in their later lives depending on their family, especially their children (Ho 2008).

In this study, participants *living with family* tended to be male and those *living only with spouse* or *living alone* tended to be female. Several studies have indicated no disadvantages in the health or mortality of older women living alone (Magaziner *et al*. 1988; Steinbach 1992). Conversely, several studies have mentioned that older men who live alone have a higher risk of mortality (Hanson *et al*. 1989; Helsing, Szklo and Comstock 1981). The reason for this difference may be related the fact that older women develop better coping strategies and contingency plans, such as using formal health services (Cafferata 1987; Tennstedt *et al*. 1990), and use of formal health services by older people living alone can help them maintain an independent lifestyle.

The present study has several strengths. First, the participants were from a national random sample of older people with a high completion rate rather than a small community sample. Second, this was a multi-wave study rather than a cross-sectional or single follow-up study. Third, we controlled for various important potential confounding factors. Fourth, survival status of each participant in the study sample was followed through 11 years of observation between 1996 and 2007. Fifth, mortality was verified through national death records by matching the citizen identification numbers of the study sample in 1996 with those of national death records rather than by other sources. Thus, survivor biases may not occur due to loss to follow-up or missing participants. Sixth, mortality risk was predicted by participant status collected in 1996, the baseline year.

The present study also has a few limitations. One limitation is that we only adjusted for the effects of confounding variables based on their status in 1996, while some confounding variables such as living arrangements and health status may change later during the follow-up period. Another limitation is that the scope of our study was confined to family support only and did not include a larger social context of social support such as community volunteering. Another is that the study did not include the combined effects of both providing and receiving family support on mortality and did not weigh the benefits of one kind of care over another. Still another limitation is that, although loneliness has been associated with mortality, we did not include it as a control variable because we did not have access to this information (Chan *et al*. 2015). Another is that although the findings of this study highlighted the benefits of providing support, it did not take into consideration the possible downsides, including disputes over how support is given and whether it was wanted or not. Finally, all the TLSA studies were self-reported and thus there may be some misclassification.

In conclusion, this study found providing family support to be associated with improved mortality in older Taiwanese people living with their families in both models. Instead of trying to do everything for older members and hiding family matters from older members, it might be more beneficial to try to engage older adults in family affairs. Their health may benefit and this would allow them to give back to those supporting them in a meaningful way. The positive attributes associated with giving back may have a more important influence on the quality of life for ageing individuals than the actual support received. Though this study did not investigate it, health-care providers may also want to encourage more able, healthier older people to do more community volunteer work to help them achieve successful ageing and longevity.
